# Measurement of attacks and interferences with health care in conflict: validation of an incident reporting tool for attacks on and interferences with health care in eastern Burma

**DOI:** 10.1186/1752-1505-8-23

**Published:** 2014-11-03

**Authors:** Rohini J Haar, Katherine HA Footer, Sonal Singh, Susan G Sherman, Casey Branchini, Joshua Sclar, Emily Clouse, Leonard S Rubenstein

**Affiliations:** Department of Emergency Medicine, St. Luke’s-Roosevelt Hospital, 1111 Amsterdam Avenue, New York, NY 10026 USA; Johns Hopkins Bloomberg School of Public Health, 615 N. Wolfe St. E7141, Baltimore, MD 21205 USA; Department of Epidemiology, Johns Hopkins Bloomberg School of Public Health, 615 N. Wolfe St. E7141, Baltimore, MD 21205 USA; Johns Hopkins Bloomberg School of Public Health, 615 N. Wolfe Street, E6543, Baltimore, MD 21205 USA; Department of International Health, Johns Hopkins Bloomberg School of Public Health, 615 North Wolfe Street, Baltimore, MD 21205 USA; Johns Hopkins Bloomberg School of Public Health, 615 N. Wolfe St. WB 602, Baltimore, MD 21205 USA; Johns Hopkins Bloomberg School of Public Health, Center for Public Health and Human Rights, 615 N Wolfe Street, E7148, Baltimore, MD 21205 USA

**Keywords:** Conflict, War, Attacks on healthcare, Health workers, Violence, Medicine, Safeguarding health, Health protection, Health and human rights, Humanitarian law

## Abstract

**Background:**

Attacks on health care in armed conflict and other civil disturbances, including those on health workers, health facilities, patients and health transports, represent a critical yet often overlooked violation of human rights and international humanitarian law. Reporting has been limited yet local health workers working on the frontline in conflict are often the victims of chronic abuse and interferences with their care-giving. This paper reports on the validation and revision of an instrument designed to capture incidents via a qualitative and quantitative evaluation method.

**Methods:**

Based on previous research and interviews with experts, investigators developed a 33-question instrument to report on attacks on healthcare. These items would provide information about who, what, where, when, and the impact of each incident of attack on or interference with health. The questions are grouped into 4 domains: health facilities, health workers, patients, and health transports. 38 health workers who work in eastern Burma participated in detailed discussion groups in August 2013 to review the face and content validity of the instrument and then tested the instrument based on two simulated scenarios. Completed forms were graded to test the inter-rater reliability of the instrument.

**Results:**

Face and content validity were confirmed with participants expressing that the instrument would assist in better reporting of attacks on health in the setting of eastern Burma where they work. Participants were able to give an accurate account of relevant incidents (86% and 82% on Scenarios 1 and 2 respectively). Item-by-item review of the instrument revealed that greater than 95% of participants completed the correct sections. Errors primarily occurred in quantifying the impact of the incident on patient care. Revisions to the translated instrument based on the results consisted primarily of design improvements and simplification of some numerical fields.

**Conclusion:**

This instrument was validated for use in eastern Burma and could be used as a model for reporting violence towards health care in other conflict settings.

**Electronic supplementary material:**

The online version of this article (doi:10.1186/1752-1505-8-23) contains supplementary material, which is available to authorized users.

## Background

Attacks on health workers and other types of interferences with healthcare services pose a considerable burden on communities and providers in conflict zones. The problem is receiving increased international attention, including publication of country-based case studies in very different contexts, such as Pakistan, Iraq, Afghanistan, Yemen and Nepal [1–8]. These studies highlight the types of attacks and interferences with healthcare including damage or destruction of health facilities, occupation and armed entry of premises, arrests, kidnappings and intimidation of health providers and patients, and attacks on and obstruction of health transports [9]. Armed conflict and other civil disturbances (which we refer to collectively as “conflict”) can lead to reductions to, or suspension of, vital medical services and denial of access to patients. Decreased access to vital medications and services can significantly impact the health of communities, particularly when the conflict and attacks on healthcare are long-standing [10]. This paper addresses the need for, and validation of, a tool that can be utilized by local health providers to report attacks toward the objective of greater protection and accountability, specifically in the setting of eastern Burma.

Local and international human rights organizations as well as UN agencies have provided valuable documentation on incidents in specific countries, but systematic tracking has been limited [11–17]. As a result, there exists a lack of data to address important questions concerning the dynamics of attacks including type of infrastructure at risk, victim profile, perpetrator profile and other potential sources of vulnerability such as time of day of incident, and mobile versus static services. Further, while most attention has been paid to attacks on international agencies, ICRC’s most recent global report found that local providers accounted for 91% of the 319 incidents, suggesting that development of a tool for use by local health providers could enable locally driven data collection to fill a significant gap in knowledge, inform protection strategies and provide a basis for accountability [18].

One country where attacks on health have been a chronic and severe problem is Burma, also known as Myanmar, where a conflict between ethnic-based armed groups and the ruling military junta has been ongoing for decades and has been characterized by major human rights abuses [19, 20]. Attacks on health services in the eastern states of Burma, and the far reaching impact on the health care system there are rarely reported on, except through the local human rights groups and organizations providing cross-border medical care. They have documented health workers beaten or jailed and patients halted at checkpoints and prevented from accessing care, among other violations of international humanitarian law (IHL) and international human rights law (IHRL) [21, 22]. These attacks have taken place against a background of governmental neglect of social and health services and especially poor health indicators among the ethnic minorities, particularly among the hill tribes along the mountainous Thai border of eastern Burma [23–26]. The maternal mortality rate is 721 per 100,000 live births in eastern Burma (covering the states of Karen, Mon and Shan, as well as two divisions - Bago and Thanintharyi), nearly three times the national rate of 240 [27]. Community-based health organizations in eastern Burma have sought to fill the gap in health services left by the government, but have been targeted for doing so. Though peace accords were signed with several armed groups in late 2011, hopes for genuine peace remain uncertain, and healthcare continues to be targeted in some regions [12].

Organizations providing health care in eastern Burma have sought a uniform and effective means for reporting these attacks. The aim of this study was to develop an easy-to-use instrument to enable more systematic reporting on such incidents. This context was identified as particularly suitable for instrument development, due to the partners’ commitment to documenting attacks and interferences with healthcare delivery as a component of their broader health systems data collection [22, 27, 28]. In developing such a tool, the authors anticipate that organized and pertinent information will permit organizations to identify and report attacks, as well as aid in prevention, protection and accountability. As victims and witnesses of attacks, local health providers and their staffs are ideally suited to act as the first line reporters of incidents. Educating local health providers to document their experiences on a standardized instrument could empower them to inform themselves of their rights and advocate on their own behalf.

**Figure 1:**
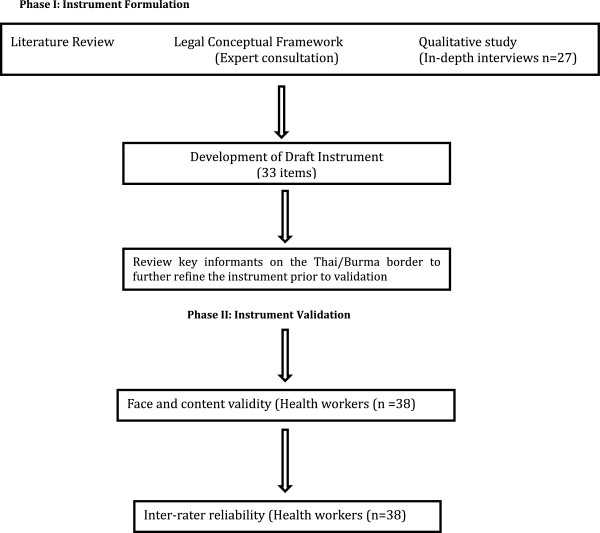
**Instrument development.**

The incident reporting form, (hereafter “the instrument”), was developed by a research team at the Center for Public Health and Human Rights at Johns Hopkins University, Bloomberg School of Public Health (JHSPH), as part of a multi-part project to address vulnerabilities of health in conflict. Phase 1 of the study included 1) a systematic literature review of recent peer-reviewed and grey literature [n = 20] documenting attacks on and interferences with healthcare occurring across conflict-affected countries [29] 2) a focused review of how international humanitarian law and human rights law bear on the problem [30] 3) qualitative in-depth interviews with supervisory health workers [n = 27] who have worked in conflict affected regions of eastern Burma [31] and 4) review of an early draft of a proposed instrument with key informants on the Thai/Burma border to further refine the instrument prior to validation (see Figure 1). The results of the first three parts of Phase 1 research were used by the research team to develop a simple but inclusive 33 item draft instrument. The items in the instrument are grouped according to four domains of violence against health care in conflict. These consist of attacks and interferences on 1) health care workers; 2) patients; 3) health care facilities and 4) health care transports. Items also cover the time, location and identity of the victims and perpetrators involved in the incident. Table 1 describes the key definitions and main domains of the instrument. Phase 2, reported on in this paper, aimed to determine the face and content validity and inter-rater reliability of the instrument. In subsequent phases, the research team plans to adapt the tool for use in other countries affected by violence against health services, and employ the technology of mobile devices to collect information on attacks on health services.

**Table 1:** **Definitions and item domains of instrument**

General terms	
Conflict	Armed conflict, both international and non-international conflicts, as well as situations that fall short of armed conflict, including internal disturbances such as political or civil violence.
Threats	Involve communicating the intention to launch an attack, inflict harm, or impede access.
Interference with Access	Includes acts that restrict the giving or receiving or health care, with or without violence, e.g. blocking entry to facilities, prevention or limitations of movement on roads or through checkpoints.
Victim-specific terms	
Health care personnel	Any person providing or attempting to provide health care or attention to a patient such as doctors, nurses, midwives, nurses’ aids, community health workers, ambulance attendants and drivers, pharmacists, and voluntary first aid providers.
Patient	Any person seeking medical care, including persons seeking care for disease or injury, routine or preventive health services such as vaccination, prenatal care, or newborn screening.
Perpetrator –specific terms	
Government Army	Persons belonging to the military branch of a state authority.
Government Police	Persons belonging to the civil force of a state or local government, responsible for the prevention and detection of crime and the maintenance of public order.
Paramilitary	Persons belonging to a group of personnel with a military structure, functioning in support of military forces of a state.
Ethnic armed Groups	Named or unspecified entities bearing weapons not on behalf of the State but on behalf of ethnic minorities within a state
Infrastructure-specific terms	
Health care facility	Any building that is known to be the site for the provision of medical services, treatment or storage of medical supplies, whether temporary/permanent or mobile, marked or unmarked.
Health care transport	Any vehicle used to transport persons in need of care or medical supplies (e.g. marked or unmarked ambulance, private car, etc.)
Medical supplies	Any items necessary for the rendering of medical diagnostic services, treatment, management or preventive services.
Incident-domains	
	Attack types:
	➢ Physical attacks on health care workers or person(s) seeking care
	➢ Physical attacks on health care facilities
	➢ Physical attacks on health care transports
	➢ Military use of health care facilities
	Threat types:
	➢ Threat directed health care worker or persons seeking care
	➢ Threat directed at health care facility (e.g., to burn it down)
	➢ Threat directed health care transports (e.g. to destroy it)
	Interference types.
	➢ Prevention of access to health worker to provide care to a patient wanting treatment
	➢ Interruption in health worker’s treatment of patient
	➢ Delay to health worker’s attempt to provide care

## Methods

This study was performed in Mae Sot, Thailand among Burmese healthcare workers. The validation process is described for a Burmese and Karen language instrument. This includes: 1) study site description, 2) translation process; 3) determining the face and content validity; and 4) ensuring inter-rater reliability of the instrument.

### Study site description

*Study Site and Partners* - The validation component was undertaken in August 2013 in the town of Mae Sot, Tak Province in western Thailand. Mae Sot is the major access point between Thailand and Burma and is within 3 km of the Burmese border town of Myawaddy. Mae Sot hosts not only a large number of Burmese migrants, but is also the headquarters of many international, national and community-based organizations that work with local communities in Burma. Several organizations operate health centers, mobile clinics, a hospital and transport services within Burma, while managing and providing administrative support from Mae Sot. JHSPH partnered with three health organizations based in Mae Sot during this study: Back Pack Health Workers Team (BPHWT), Burma Medical Association (BMA) and Karen Department of Health and Welfare (KDHW). BMA and KDHW consider themselves to be governmental health organizations in exile but all three groups are registered as non-governmental organizations in Thailand. The cross-border nature of the organizations’ work, in predominantly opposition held areas, is supported by local ethnic Burmese health workers who are able to work with local councils, camp and community leaders. This is key to their success and ability to reach areas historically inaccessible to humanitarian assistance. All organizations work to provide health care in accordance with principles of medical impartiality, although their actual or perceived affiliation to armed groups has increased their vulnerability [32].

All three organizations primarily serve communities in rural eastern Burma with basic healthcare services including primary care, vaccination, management of acute illness, simple surgical procedures, and reproductive health and midwifery services. The BPHWT serves 221,000 patients with over 95 mobile health teams that travel to remote and conflict regions to address local needs [28]. KDHW serves more than 100,000 internally displaced Burmese and BMA supports more than 40 clinics that serve 180,000 Burmese throughout eastern Burma. While the government of Burma now provides limited support for healthcare in this region, these organizations have been the primary providers of health services for a large and diverse population, particularly during the most volatile periods of eastern Burma’s ethnic conflict. These organizations were chosen for this study because they are the chief sources of medical care for communities in eastern Burma, expressed interest in improving their ability to collect data on attacks on their health workers and clinics, and through their headquarters in Mae Sot, are accessible to research institutions.

*Study population* - Health care workers from BPHWT were invited to participate in Phase 2 of the study, but staff from all three organizations actively took part in Phase 1. BPHWT workers with a wide range of skills and experience were chosen to participate in the validation because field experience during decades of chronic conflict ensured that participants have some knowledge of attacks and/or interference with health relevant to the instrument and the logistical and administrative structure of the biannual return of health workers to Mae Sot for training was well-suited to a timely and efficient study. BPHWT medics are community health workers trained in primary care and basic surgical techniques but do not have formal medical education. BPHWT field workers do not include health professionals such as nurses and doctors. The opportunity to partner with health providers that include staff with professional training was not possible due to the absence of such providers in this region and restrictions on access and security. Clinical and administrative supervisors at BPHWT headquarters identified study participants based on their field experience, willingness to discuss relevant topics and availability for discussion groups from among nearly 70 health workers who had returned from their primary mobile medical sites in Burma in August 2013. Suggested participants were requested to participate by their supervisors and informed that involvement was entirely voluntary and that they could decline without any consequences for their employment. Eligibility criteria required that participants be over 18 and medics with BPHWT.

*Demographic characteristics* - We held five discussion groups with group sizes of 6–9 aimed at ensuring meaningful conversation, with n = 38 ensuring saturation was met. Participants were 18 women and 20 men. Participants’ years of experience ranged between 0.5 and 10 years with a mean of 3.6 years of experience in their respective specialties. Forty-seven percent of participants were ethnic Karen but only 2 participants (5%) required the Karen translation of the instrument; all other participants used the Burmese form. The participants came from the following states: Karen, Rakhine, Shan, Kachin, and Kayan States. Participants included Field in-Charges (FiCs) [9], Maternal & Child Health Program health workers (MCH) [12], Medical Care Program health workers (MCP) [6], Community Health Education and Prevention Program workers (CHEPP) [7], and general Health Workers (HW) [4]. “Field in-Charges” are lead health workers in a major target area with one or more mobile health teams; their role is to manage health workers on the mobile health teams in their respective field area as well as to liaise with the administrative and programmatic staff in Mae Sot. MCH and MCP health workers provide maternal/child and primary care (six main diseases and war trauma injuries) respectively. CHEPP health workers provide preventative health services such as health education as well as clean water and sanitation systems to schools and communities and general health workers assist Field in-Charges and other workers with clinical duties.

### Translation and back translation

The English version of the instrument developed in Phase 1 was translated into Burmese and Karen by native Burmese and Karen bilingual translators. These versions were then back-translated into English by translators who had not seen the original English version. The first author compared the back-translated copy to the original English version to identify incongruities. The Burmese and Karen translations were then adjusted with corrective re-translation if necessary.

### Measures and analysis

This study utilized qualitative and quantitative evaluation methods to ensure robust testing of the instrument. The instrument was validated through face validity, content validity and inter-rater reliability.

*Face and Content Validity* - Face validity is the qualitative assessment that a survey reflects what it purports to measure [33]. Content validity measures whether the content of the tool is appropriate, relevant and correctly addresses the intention of the instrument [34]. Five discussion groups of 6–9 participants met with investigators for 4–4.5 hour sessions over three days. Upon conducting the study, saturation of ideas and responses was reached after 30 participants, but another discussion group was held to ensure completeness, leading to a total of 38 participants.

A trained Burmese or Karen translator with knowledge of human rights issues was present throughout all the sessions. The translator’s role was to directly translate investigator and participant comments and, as necessary, interpret the comments for better comprehension. Both translators worked previously in social justice organizations and had the relevant vocabulary and context to translate the content of the study process. Prior to the discussion groups, study investigators who had assisted with instrument development, and translators held briefings to review the content, language and goals of the study.

Item-by-item discussion was conducted utilizing a pre-written open-ended discussion guide. The discussion guide was structured to concentrate on the following key areas to determine face and content validity: 1) design of survey (layout, order, length); 2) language (translation, clarity, vocabulary, brevity and focus); 3) applicability and specificity of the items. Questions were asked about each domain, (i.e. attacks on health facility domain). Participants then were asked about each item within the domain, (i.e. impact on facility). Open-ended initial questions on each domain and each item were followed-up with more specific queries to clarify responses and probe any confusing issues.

Data analysis followed a constant comparative method for qualitative data categorization and analysis [35]. The first two authors participated in data analysis and categorization; the first author coded data, which was re-checked and discussed by members of the study team. Discussion notes were reviewed after each session and emerging themes and key points were documented.

*Inter-rater reliability* - Inter-rater reliability refers to the ability of different participants to consistently complete the instrument with the correct information given the same initial data [36]. The instrument was completed twice by participants using two different simulated incident scenarios and inter-rater reliability was assessed via scoring of participants’ completed reports. For authenticity, the scenarios were based on attacks and interferences similar to those previously documented in eastern Burma. Scenario 1 concerned a health worker who was beaten and had his supplies confiscated while traveling through the forest from one mobile clinic to another. Scenario 2 was the account of a medical clinic that was attacked, burned down and subsequently forced to close. Detailed information on dates, time, location and witnesses were provided for both scenarios. The translator presented the scenarios verbally and any questions were answered based on the script provided. Participants were allowed as much time as they required to complete the instrument. Subsequent to the completion of the simulated scenarios, participants were asked to provide feedback on their experience of completing the instrument.

For the simulated scenarios, time to completion was recorded for each group. Completed instruments were coded and graded for percent agreement compared with an answer sheet developed by the investigators. The completed instruments were evaluated under two criteria: 1) comprehension and reporting of the “essential facts” of the incident and 2) item validation wherein each item on the instrument was analyzed discretely. The “essential facts” were defined as the events of importance in the incident that would reveal who was involved, where and when the incident occurred and what happened, including the impact of the incident.

After the validation and analysis, the survey was finalized to remove any confusing language, correct translation errors, and improve the design and layout. The instrument is included as Additional file 1. Local health groups including BPHWT, KDHW, and BMA have versions in Burmese and Karen finalized for field use.

### Human subjects protection

The Institutional Review Board (IRB) of JHSPH and a local review board convened in Mae Sot, Thailand, approved this study. To guarantee the confidentiality and security of participants, no individual identifiers were used. All participants provided informed consent prior to participation in the discussion groups, which were conducted by the first two authors in the presence of skilled local interpreters. Verbal consent was used to further safeguard participant privacy and security.

## Results

### Validity testing

Face validity was ascertained through structured discussion groups. Participants gave recommendations to enhance language, translation, and design layout that were subsequently incorporated into the instrument. The participants agreed that the instrument was comprehensible and addressed their subjective understanding of what constituted an attack or interference with healthcare in the setting of eastern Burma. Their recommendations centered on making the form shorter, visually more appealing and removing individual number counts for violation categories. There was general consensus among participants that the security conditions were such that the instrument could be used in the field without significant risk both now when there was some relative peace and potentially in the future during times of more active conflict. The few participants who did express security concerns acknowledged that they regularly carry confidential medical information, which already poses inherent risks that are not substantially increased by this additional form. Several participants advised methods to make the form less conspicuous, such as writing it only in local script rather than in local and English versions on the same form, adding it to the back of their pre-printed clinical registers rather than as a separately printed sheet, or keeping one form blank and writing the relevant responses for each incident on blank note paper kept separately.

Content validity was also ascertained during the course of the structured discussion groups. Participants agreed that individual items in the instrument were appropriate to the setting in which they worked and met with their experiences and understanding of attacks and interferences with health care. Disagreements with items were minor and focused on wording rather than content. In discussion of the four domains of health care workers, patients, facilities and transports, attacks or interference with health transports was the least applicable, as the majority of health workers’ experiences involved delivering care on foot. However, most health workers did see the relevance of the section in terms of movement of patients or medical supplies by private car, which would fall within the scope of a healthcare transport.

### Reliability testing

Inter-rater reliability was ascertained through the completion of the instrument based on the two simulated scenarios. All 38 participants filled out the instrument for both scenarios. There was a significant difference between completion times for the two scenarios (*p* = 0.011); mean completion time for the scenarios per group was 22.6 minutes for Scenario 1 and 18 minutes for Scenario 2 (Table 2). The completed instruments were analyzed to provide quantifiable answers to the question: Can participants reliably use the instrument to capture the “essential facts” of the instrument (those events that reveal a complete account of the incident including who was involved, when and where it occurred and what happened, including impact). Scoring was based on correctly addressing these essential facts utilizing both the standard value fields (checkboxes) and the narrative description. There were three steps associated with reliability testing: 1) Each individual response was graded as accurate or inaccurate. Inaccurate responses would be individually evaluated as “partially correct, “incorrect” or “blank” based on the relevance of each specific item to the essential facts. 2) each participant would receive a final score of “acceptable” if all essential facts were reported correctly or there were 2 or fewer errors categorized as either “partially correct” or “blank.” Incorrect responses were not considered acceptable for the essential facts. 3) Investigators agreed that the instrument would be considered successfully validated if 50% of all participants received a final score of “acceptable”. Though little direct evidence exists on benchmarks in qualitative validations, this threshold was based on investigator discussions of the importance of accurate data balanced against the necessity to allow for some inaccuracies given the complexity of human rights reporting. Using a standard normal distribution in evaluating responses, authors concluded that if 50% of the participants are correctly able to utilize the instrument to report incidents of attack or interference with healthcare in the validation study, then there is a high probability that the large majority of users in the field will be able to give useful details about relevant incidents. In any reporting of this nature, there is a risk that inaccuracies in reporting could cause further violence or damage fragile relationships. Given that risk, the responsibility of the organizations collecting this data is to review the reports and determine whether there is sufficient basis for the report before engaging in wider dissemination or action.

**Table 2:** **Completion times for quantitative scenarios (in minutes)**

Group #	Scenario 1	Scenario 2
**1**	**25**	**20**
**2**	**20**	**18**
**3**	**25**	**17**
**4**	**23**	**20**
**5**	**20**	**15**
**Average**	**22.6**	**18**

For Scenario 1: 58% of participants correctly addressed all six of the essential facts of the scenario using only the checkboxes, (i.e. *two* army affiliated perpetrators beat *one* health worker at a *checkpoint* in *May 2013* and *stole* supplies). An additional 28% were able to provide all of the essential facts with two or fewer individual responses categorized as “partially correct” or “blank”- these were most commonly the specific numbers of perpetrators or number of victims. For Scenario 2: 45% of participants were able to correctly address the essential elements of the incident using only the checkboxes (i.e. *1* medical clinic was *burnt* by *army* perpetrators with *no* casualties and the facility has since *closed*.) 37% had 2 or fewer errors that were categorized as “partially correct” or “blank”, primarily in the field to identify that the facility had closed (see Table 3). In total, 86% of the participants on Scenario 1 and 82% on Scenario 2 reported results in the range considered acceptable (“essential facts” reported correctly with 2 or fewer errors).

**Table 3:** **“Essential facts” reporting**

Component	Scenario 1	Scenario 2
**100% of essential facts**	22 (57.9%)	17 (44.7%)
**1-2 errors**	11 (28.9%)	14 (36.8%)
**3-6 errors**	5 (13.2%)	7 (18.4%)
**Total “Essential Facts” in Acceptable Range (including 1–2 errors)**	**33 (86.8%)**	**31 (81.5%)**

In addition to the above analysis of the scenario as a whole, each item was also discretely evaluated to ensure validity for field use (Tables 4 and 5). For Scenario 1: Greater than 95% correctly reported on Section A (Who) and Section B (When and Where) under items for identification, time and location (Table 4). 76% of respondents correctly reported the identity/affiliation of the person reporting on the incident and 82% correctly reported the identity/affiliation of the perpetrator. The primary notable error was in quantifying the impact of the incident on patient care (21% correctly completed), i.e. was treatment delayed, interrupted or prevented. Participants also had difficulty in reporting numerical data (i.e. 58% of participants correctly documented number of perpetrators on Scenario 1). Other errors included coding within the incorrect section (health worker information in the patient section) and blank sections, particularly for narrative data (44% recorded narrative data).

**Table 4:** **Item validation scenario 1**

Scenario A/1		Correct	Partial	Incorrect	Skipped	Correct %	Partial %	Incorrect %	Skipped %
Who	Field ID#	38	0	0	0	100%	0%	0%	0%
	Identification	37	0	0	0	97%	0%	0%	0%
	Person reporting	29	0	0	9	76%	0%	0%	24%
	Perpetrator/Accused	31	1	1	5	82%	3%	3%	13%
	Number of perpetrators	22	0	0	14	58%	0%	0%	37%
When and where	Date of incident	38	0	0	0	100%	0%	0%	0%
	Time of day	37	0	0	1	97%	0%	0%	3%
	Location of incident	38	0	0	0	100%	0%	0%	0%
	GPS coordinates	0	0	0	0	0%	0%	0%	0%
	Type of location	36	0	0	2	95%	0%	0%	5%
Attack/interference on health care personnel	Was there an attack	32	4	1	1	84%	11%	3%	3%
	Type of attack	36	0	0	2	95%	0%	0%	5%
Attack/interference on patient	Was there an attack	31	2	5	0	82%	5%	13%	0%
	Type of attack	n/a	n/a	n/a	n/a	n/a	n/a	n/a	n/a
	Was access to health care prevented/delayed/how long?	8	0	0	28	21%	0%	0%	74%
Attack/interference on health care facility	Was there an attack	35	1	1	0	92%	3%	3%	0%
	Name of facility	n/a	n/a	n/a	n/a	n/a	n/a	n/a	n/a
	What happened to the facility	n/a	n/a	n/a	n/a	n/a	n/a	n/a	n/a
	Impact of the clinic	n/a	n/a	n/a	n/a	n/a	n/a	n/a	n/a
	Label or emblem on the health care facility	n/a	n/a	n/a	n/a	n/a	n/a	n/a	n/a
Attack/interference on health care transport	Was there an attack	38	0	0	0	100%	0%	0%	0%
	Type of attack	n/a	n/a	n/a	n/a	n/a	n/a	n/a	n/a
	Impact of the transport	n/a	n/a	n/a	n/a	n/a	n/a	n/a	n/a
	Was there a label or emblem on the health care transport	n/a	n/a	n/a	n/a	n/a	n/a	n/a	n/a
Narrative	Description of the attack in narrative format	17	0	0	19	45%	0%	0%	50%

**Table 5:** **Item validation scenario 2**

Scenario D/2		Correct	Partial	Incorrect	Skipped	Correct %	Partial %	Incorrect %	Skipped %
Who	Field ID#	38	0	0	0	100%	0%	0%	0%
	Identification	38	0	0	0	100%	0%	0%	0%
	Person reporting	11	13	1	13	29%	34%	3%	34%
	Perpetrator/Accused	28	5	4	1	74%	13%	11%	3%
	Number of perpetrators	22	0	0	14	58%	0%	0%	37%
When and where	Date of incident	38	0	0	0	100%	0%	0%	0%
	Time of day	38	0	0	1	100%	0%	0%	0%
	Location of Incident	38	0	0	0	100%	0%	0%	0%
	GPS coordinates	n/a	n/a	n/a	n/a	n/a	n/a	n/a	n/a
	Type of location	38	0	0	0	100%	0%	0%	0%
Attack/interference on health care	Was there an attack	37	0	1	0	97%	0%	3%	0%
	Type of attack	0	0	0	0	n/a	0%	0%	0%
Attack/interference on patient	Was there an attack	37	0	1	0	97%	0%	3%	0%
	Type of attack	n/a	n/a	n/a	n/a	n/a	n/a	n/a	n/a
	Was access to health care prevented/delayed/how long?	n/a	n/a	n/a	n/a	n/a	n/a	n/a	n/a
Attack/interference on health care facility	Was there an attack	34	0	0	4	89%	0%	0%	11%
	Name of facility	23	1	0	14	61%	3%	0%	37%
	What happened to the facility	31	1	0	6	82%	3%	0%	16%
	Impact of the clinic	27	0	0	11	71%	0%	0%	29%
	Label or emblem on the health care facility	22	1	1	13	58%	3%	3%	34%
Attack/interference on health care transport	Was there an attack	33	0	0	5	87%	0%	0%	13%
	Type of attack	n/a	n/a	n/a	n/a	n/a	n/a	n/a	n/a
	Impact of the transport	n/a	n/a	n/a	n/a	n/a	n/a	n/a	n/a
	Was there a label or emblem on the health care transport	n/a	n/a	n/a	n/a	n/a	n/a	n/a	n/a
Narrative	Description of the attack in narrative format	8	0	0	30	21%	0%	0%	79%

For Scenario 2, 100% correctly reported on Section A and B under identification, time and location. 58% correctly reported the identity/affiliation of the person recording the information and 74% correctly reported the perpetrator identity (Table 5). Greater than 95% completed all of the correct sections. Focusing on the health facility section, 60% correctly reported the facility identification, 82% correctly reported the specific circumstances of the incident (facility burned) and 71% reported correctly on the impact to the facility (remains closed). Of note, 58% were able to state whether there was a label/emblem on the facility and only 21% recorded any narrative data (see Table 5). Results also highlighted that participants were more likely to skip an item, compared to answering it incorrectly. For instance, in Scenario 2, 58% of participants answered the item “was there a label or emblem on the facility” correctly, 3% answered incorrectly and 34% left the field blank. Domains on healthcare workers, patients and healthcare facilities were correctly completed (not attacked) in 80% or greater of the completed instruments.

Feedback by participants after completion of the scenarios indicated that they found the instrument easier to navigate after having used it for the first time on Scenario 1. They also noted that some sections were unclear, most notably the impact on patients (i.e. the distinction between a delay in treatment, an interruption to treatment, and the prevention of treatment). On reviewing the completed items, it was noted that respondents had difficulty accurately recording the number of victims and/or perpetrators involved in an incident. In response investigators revised the instrument by changing the open-ended numerical fields to closed-ended response choices, such that respondents now check a box indicating “0”, “1-5”, “6-10”, “11 or more” and “don’t know”.

## Discussion

This study validated a newly developed instrument to capture incidents of attacks or interference on health in eastern Burma. The findings of the qualitative and quantitative portions of the study suggest that the instrument is an effective and reliable instrument for health workers to report incidents of attack or interference with their daily work. Although limited to one setting, the instrument represents a first step toward more systematic reporting of attacks or interference with health and can provide a model for reporting attacks or interferences on health in other conflict regions.

Participants found the instrument relevant and applicable. They determined that the instrument appropriately reflects the intended outcome (face validity) and that it has an appropriate breadth and depth of information without being excessively cumbersome (content validity). Participants were able to comprehend the details of each domain but reported some unfamiliarity with healthcare transports in their own context. Despite this hurdle they were able to give examples of transports and discuss the importance of including a transport domain. Within each domain individual items were discussed and readily understood. Respondents did express the view that an element of subjectivity exists in interpreting some forms of attacks, such as interrogation and threats. After discussion, investigators simplified the numerical data fields to reduce error (from open to closed-ended answer choices) but retained the same categories (i.e. interrogation, threaten etc.) in the expectation that despite some subjectivity, the fields continue to provide a useful means of capturing those interferences that do not constitute violent attacks, but still represent important violation categories. One of the key goals of a standardized reporting tool is to develop some quantifiable data so it was important to retain the numerical fields while making them easier to use.

On evaluation of participant responses based on two scenarios, investigators found that participants were able to appropriately give the relevant information and provide insight into the extent of damage from an attack or interference with healthcare. Investigators agreed prior to field testing that 50% of participants accurately reporting the “essential facts” would assure that the instrument was valid based on the purposes for which the data collection would be used. This instrument is intended to provide an overall picture of the numbers and types of incidents, as well as a basis for further investigation to verify incidents of attacks or interference with healthcare. To determine whether an act constitutes a violation of international law, such that accountability procedures should be considered, more rigorous investigation and verification would be required. For this baseline data collection and reporting mechanism, however, some inaccuracy in reporting on incidents would be acceptable. The authors concluded that this level of accuracy in reporting is appropriate to develop a better understanding of the frequency of incidents, particularly non-violent interferences and their impact on health care delivery. This could inform protection strategies in health delivery and be used for national and international advocacy. The results that 86% of participants responding to Scenario 1 and 82% of participants responding to Scenario 2 reported “acceptable” accounts of the incidents indicates that the instrument is a reliable tool for reporting the numbers and types of attacks and interferences on healthcare in this context.

The time required to complete the tool was significantly less for Scenario 2 than for Scenario 1, and we predict that with further instruction and practice, the instrument will take less time to complete. Though respondents indicated that Scenario 2 was easier to complete, it is noted that more respondents scored “acceptable” on Scenario 1 (86% vs. 81%). The difference between these percentages is not statistically significant (p = .7). Nevertheless, this trend may be the result of the increased difficulty level of Scenario 2 as compared to Scenario 1 or other factors that have not been fully evaluated.

Item-by-item evaluation revealed that most items on the instrument were correctly completed. Investigators also observed that participants left some fields blank. One disadvantage of a closed-ended questionnaire is the possibility that respondents leave the response blank, making it unclear to investigators whether respondents did not understand the question, did not find an appropriate response within the closed-ended answer choices, or did not read through the survey thoroughly. Investigators have addressed this concern by revising the answer choices to include “unknown” fields among the answer choices, and the written instructions accompanying the survey set out that all fields should be completed.

On item-by-item validation, it was noted that only 21% of participants correctly completed the impact questions concerning treatment delay vs. interruption. The authors reviewed the impact questions closely and concluded that ascertaining the health impact requires additional data beyond the factual documentation of incident details. As the distinction between interruption of care and delay can be ambiguous (i.e. every interruption causes a delay), responding to these questions may be difficult. However, we expect that in other settings some additional information would be available and would reveal valuable information relevant to the impact of the incident. It is important to note that participants were guided through an item-by-item discussion of the instrument, but were not given formal training or definitions to aid completion of the instrument. Our findings suggest that training would assist in improving the reliability of responses and the comprehension of more difficult questions. We expect that the minor changes in the layout and design, and additional training prior to use in the field will better equip health workers in the future to complete the instrument in its entirety.

This validation process had several limitations. The instrument validation was conducted in one context on the Thai/Burmese border among Burmese health workers. Participants are qualified as “medics” by unofficial training programs and work experience. Many of the participants lack formal education beyond the grade school level and none have formal medical education. Conducting the study with formally trained clinicians in other settings may have yielded different results of the validation study. This may have biased the validation towards less trained personnel, rather than highly trained health workers such as physicians. Conversely, it is likely that in settings with more formally educated medical personnel the form will be filled out with similar if not greater accuracy.

As the instrument was translated from English into both Burmese and Karen, some language in both the reporting form may have been misunderstood. Investigators have attempted to minimize translation errors via verifying a back-translation and ensuring that discussion groups provided feedback on the translation.

Given the lack of healthcare transports used in this setting, investigators did not provide a scenario on health transport attacks. Though this omission may represent an incomplete validation of these items, the strong results in the other attack categories indicate that the transport section of the instrument, with similar answer fields, is reliable.

Responses and attitudes towards simulated scenarios in the safe environment in Mae Sot might be different from what might happen in the field, where stresses from insecurity could affect reporting. The majority of participants said that they felt safe using this form but also had thoughts on how to make the form less conspicuous in the field because possessing this data may pose some risk during crossing of checkpoints or raids of the clinics. Security is a major consideration in the utilization of this instrument and must be determined by individual organizations in collaboration with local health workers.

The validation was limited to participants chosen by administrators from one organization in a local context, potentially introducing bias based on their geographical, organizational or personal beliefs. However, participants were drawn from all areas of Burma where BPHWT works, and the responses revealed that diverse views were obtained from this study, limiting this bias. Monitoring and evaluation of day-to-day field use of this instrument, ideally, in diverse settings, would lead to improvements of the instrument.

Meetings were held with BMA, KDHW and BPHWT senior staff and data personnel to discuss the results and revisions to the instrument. The organizations indicated that they considered the form practical and relevant for their health workers to collect information in the field. Participants and administrators felt secure carrying the instrument for practical field use.

## Conclusion

To our knowledge, there has been no validated incident reporting form for health workers and health organizations to report violations involving healthcare, particularly in eastern Burma.

It is expected that this instrument can serve as a model to be adapted, with changes based on differences in contexts, to other settings. Wide adoption of such an instrument could achieve greater local awareness of the incidence of attacks in specific locations, a global database of events, and the basis for action to prevent attacks and hold perpetrators accountable. The data collected by this instrument goes beyond typical security incident reporting forms, by capturing both violent and non-violent forms of interference with healthcare, such as confiscation of medicines, delays at checkpoints, obstruction of access, and intimidation and threats. Research in this setting indicates that such interferences are more frequent than violent events, and have negative consequences for access to and delivery of healthcare [32]. Increased reporting of non-violent events can assist health organizations in assessing the frequency and impact of incidents that health workers may have previously considered an ‘everyday event’. The reporting form is also tied to international human rights and humanitarian law. Creating an evidence base is crucial to an understanding of the role of non-violent interferences in curtailing health access and violations of the right to health in chronic conflict settings.

As frontline workers, human rights organizations, NGOs and others in conflict areas seek to improve the health of their communities. This reporting form has the potential to empower local health groups to understand their human rights and provide them with a simple effective means of reporting violations within their community and at the national and international level. Although this instrument was validated in the context of eastern Burma, it can provide a model for collecting data in other settings, ensuring a better evidence base from which to advance and advocate for the better protection and respect of healthcare in times of conflict.

In the setting of eastern Burma, investigators have developed an online platform using the MagPi website that partner Burmese organizations could adopt to collate their information [37]. The MagPi system allows for mobile phone entry and transmission of information as well as real-time data retrieval capabilities in a secure online database. Investigators expect that a mobile phone platform, which is not practicable at present in eastern Burma, will be of additional benefit in other settings where mobile data collection is more feasible. A global template that can be adapted for use in other contexts to document attacks on and interferences with healthcare can be accessed by contacting the corresponding author.

## Electronic supplementary material

Additional file 1: **Attacks & interferences involving healthcare: incident reporting form.** (DOCX 88 KB)
