# Supplementary material for: Measurement of attacks and interferences with health care in conflict: validation of an incident reporting tool for attacks on and interferences with health care in eastern Burma
Source: Confl Health. 2014 Nov 3;8:23. doi: 10.1186/1752-1505-8-23 (PMC4232629; doi:10.1186/1752-1505-8-23)
Supplement: Supplementary file 1 — Additional file 1: Attacks & interferences involving healthcare: incident reporting form. (DOCX 88 KB) [file 13031_2014_144_MOESM1_ESM.docx]

***Attacks & Interferences involving Healthcare: Incident Reporting Form***

**Instructions**

*Please use the following instructions when reporting on any attacks on or interference with healthcare.*

- Only collect information about attacks or interferences involving patients, health care personnel, clinics or vehicles.
- This information is important to report so that we have a better understanding of the problems that health care personnel and patients suffer, and who is responsible.

**Filling out the form:**

- Only fill out if **safe and secure** to do so. (Names, locations or other sensitive information should be kept **confidential and secure**.)
- Report every incident separately on a new form ensuring all relevant information is included.
- Boxes should be filled with a cross mark (X).
- **If you do not know** a piece of information, **always select ‘unknown’**.
- Select **only one response unless otherwise indicated**.
- If you have any **additional evidence** (e.g. photos or video), please include in **Section H**.

**You need to provide information on:**

- **Who** was involved in the incident? **Where** was the incident?
- **What** happened? **When** was the incident?

Please write your report carefully including information on **all sections**.

| SECTION A: Who | | | | |
| --- | --- | --- | --- | --- |
| **A.1** | **Facility/Field ID#:** | \|  \|  \|  \|  \| \| --- \| --- \| --- \| --- \| \|  \|  \|  \|  \| | | |
| **A.2** | **Who reported the information on this incident?:** | Were you the victim?  Were you the witness?  Did you get information directly from a victim or eyewitness? | | |
|  |  | Other ***(please* *specify****)* | |  |
|  | **A.2.1.** [***If other to A.2*], please specify:** _____________________________________ | | | |
| **A.3** | **Who was attacked or interfered with in this incident? (*select ALL that apply*):** | Patient  Health worker  Clinic | Health vehicle  Unknown | |
| **A.4** | **Who is the accused in this incident? (*select ALL that apply*):** | Government army  Government Police  Paramilitary | Ethnic armed groups  Other ***(please* *specify)*** | |
|  | **A.4.1. [*If other to A.4*], please specify:** ___________________________________________________ | | | |
| **A.5** | **Specify the number of perpetrators (*If unknown, please write ‘unknown’*): ________________________***______* perpetrators | | | |

| SECTION B: When and where | | | | |
| --- | --- | --- | --- | --- |
| **B.1** | **Date of incident (*approximate*):** | ____ /____ /________ (DD/MM/YYYY) | | |
| **B.2** | **Time of Day:** | Morning  Afternoon  Evening  Night Unknown | | |
| **B.3** | **Location of incident (*If unknown, write ‘unknown’*)** | *Country:___________________________ State: ___________________________ Village:____________________________ District:__________________________* | | |
| **B.4** | **GPS coordinates:** | *X Coordinate*:_______________________ *Y Coordinate*:______________________ | | |
| **B.5** | **Type of location**  **(*select ALL that apply*):** | Clinic  Mobile clinic  Private home | Health care vehicle  Bush/Forest  Checkpoint  IDP/Refugee Camp | Pharmacy  Road  Unknown  Other ***(please* *specify)*** |
| **B.5.1.** [***If other to B.5*], please specify:** ______________________________________________ | | | | |

| SECTION C: Attack/interference on health care worker | | | | | | | | |
| --- | --- | --- | --- | --- | --- | --- | --- | --- |
| **C.1** | **Was there an attack or interference involving health care worker?** | | Yes  No 🡪 ***skip to D.1***  Unknown🡪 ***skip to D.1*** | | | | |  |
| **[*If yes to C.1*], please identify the number of health care personnel: (*If the information is unknown, please select ‘unknown’. Please do not leave any questions blank.)*** | | | | | | | |  |
| **C.2** | **Killed** | 0 | | 1-5 | 6-10 | 10 or more | Unknown | |
| **C.3** | **Shot** | 0 | | 1-5 | 6-10 | 10 or more | Unknown | |
| **C.4** | **Beaten** | 0 | | 1-5 | 6-10 | 10 or more | Unknown | |
| **C.5** | **Arrested** | 0 | | 1-5 | 6-10 | 10 or more | Unknown | |
| **C.6** | **Kidnapped** | 0 | | 1-5 | 6-10 | 10 or more | Unknown | |
| **C.7** | **Raped/GBV** | 0 | | 1-5 | 6-10 | 10 or more | Unknown | |
| **C.8** | **Tortured** | 0 | | 1-5 | 6-10 | 10 or more | Unknown | |
| **C.9** | **Interrogated** | 0 | | 1-5 | 6-10 | 10 or more | Unknown | |
| **C.10** | **Threatened** | 0 | | 1-5 | 6-10 | 10 or more | Unknown | |
| **C.11** | **Forced to pay a bribe** | 0 | | 1-5 | 6-10 | 10 or more | Unknown | |
| **C.12** | **Had medical supplies taken** | 0 | | 1-5 | 6-10 | 10 or more | Unknown | |

| SECTION D: Attack/interference on patient | | | | | | | | | | | | | | | | |  |  |
| --- | --- | --- | --- | --- | --- | --- | --- | --- | --- | --- | --- | --- | --- | --- | --- | --- | --- | --- |
| **D.1** | **Was there an attack or interference involving a patient?** | | | | | Yes  No 🡪 ***skip to E.1***  Unknown🡪 ***skip to E.1*** | | | | | | | | | |  |  |  |
| **[*If yes to D.1*], please identify the number of patients (*If the information is unknown, please select ‘unknown’. Please do not leave any questions blank.)*** | | | | | | | | | | | | | | | |  |  |  |
| **D.2** | **Killed** | | | 0 | | | | 1-5 | 6-10 | | | 10 or more | | Unknown | | |  |  |
| **D.3** | **Shot** | | | 0 | | | | 1-5 | 6-10 | | | 10 or more | | Unknown | | |  |  |
| **D.4** | **Beaten** | | | 0 | | | | 1-5 | 6-10 | | | 10 or more | | Unknown | | |  |  |
| **D.5** | **Arrested** | | | 0 | | | | 1-5 | 6-10 | | | 10 or more | | Unknown | | |  |  |
| **D.6** | **Kidnapped** | | | 0 | | | | 1-5 | 6-10 | | | 10 or more | | Unknown | | |  |  |
| **D.7** | **Raped/GBV** | | | 0 | | | | 1-5 | 6-10 | | | 10 or more | | Unknown | | |  |  |
| **D.8** | **Tortured** | | | 0 | | | | 1-5 | 6-10 | | | 10 or more | | Unknown | | |  |  |
| **D.9** | **Interrogated** | | | 0 | | | | 1-5 | 6-10 | | | 10 or more | | Unknown | | |  |  |
| **D.10** | **Threatened** | | | 0 | | | | 1-5 | 6-10 | | | 10 or more | | Unknown | | |  |  |
| **D.11** | **Forced to pay a bribe** | | | 0 | | | | 1-5 | 6-10 | | | 10 or more | | Unknown | | |  |  |
| **D.12** | **Had medical supplies taken** | | | 0 | | | | 1-5 | 6-10 | | | 10 or more | | Unknown | | |  |  |
|  | |  | | |  | |  | | |  | | |  | |  | | |  |
| SECTION E: Attack/interference on clinic | | | | | | | | | | | | | | | | | | |
| **E.1** | | **Was a clinic interfered with or attacked?** | Yes  No | | | | | | | | Unknown | | | | | | | |
| **E.2** | | **Name of the clinic (*If unknown, please write ‘unknown’*):** | **________________________________________________** | | | | | | | | | | | | | | | |
| **E.3** | | **Select the statements that best describe what happened to the health care facility (*select ALL that apply*):** | Clinic shelled/burnt/fired upon  Medical supplies taken or destroyed | | | | | | | | Clinic taken over by the armed actors  Armed actors prevented entry into clinic | | | | | | | |
| **E.4** | | **What was the impact on the clinic?** | Clinic open  Clinic closed  Unknown | | | | | | | | | | | | | | | |
| **E.5** | | **Was there a label or emblem on the clinic?** | Yes ***(please specify)***  No **🡪 *skip to F.1***  Unknown **🡪 *skip to F.1*** | | | | | | | | | | | | | | | |
| **E E.5.1.** [***If yes to E.5*], please specify:** __________________________________________________ | | | | | | | | | | | | | | | | | | |

| SECTION F: Attack/interference on health care vehicle | | | |
| --- | --- | --- | --- |
| **F.1** | **Was a health care vehicle interfered with or attacked?** | Yes ***(please specify)***  No **🡪 *skip to G.1***  Unknown **🡪 *skip to G.1*** | |
| **F.2** | **Select the statements that best describe what happened to the health care transport (*select ALL that apply*):** | Health care vehicle shelled/burnt/fired upon  Delayed beyond time required for a vehicle search | Medical supplies taken or destroyed  Other |
| **F.3** | **Is the health care vehicle functioning? :** | Yes  No  Unknown | |
| **F.4** | **Was there a label or emblem on the health care vehicle?** | Yes ***(please specify)***  No **🡪 *skip to G.1***  Unknown **🡪 *skip to G.1*** | |
| **F.4.1.** [***If yes to F.4*], please specify:** ____________________________________________________ | | | |

| SECTION G: Impact of the attack | | | | |
| --- | --- | --- | --- | --- |
| **G.1** | **Was access to health care prevented?** | Yes | No | Unknown |
| **G.2** | **Was access to health care interrupted?** | Yes | No | Unknown |
| **G.3** | **Was treatment of patients delayed?** | Yes ***(please specify)*** | No **🡪 *skip to H.1*** | Unknown **🡪 *skip to H.1*** |
|  | **G.3.1.** [***If yes to G.3*], please specify:** ____________________________________________________ | | | |

**H.1. Please describe any attack or interference reported on this form using your own words (when, where, what, and by whom). Avoid using personal names, if necessary, to maintain safety and security. Provide as much detail as possible.**

**END OF FORM**
